# Effectiveness of voluntary PCR testing against COVID-19 spread in remote Japanese islands

**DOI:** 10.1017/S0950268826101289

**Published:** 2026-03-24

**Authors:** Moto Kimura, Yusuke Asai, Shinya Tsuzuki, Yosuke Shimizu, Yukari Uemura, Yurika Tanaka, Junko Terada-Hirashima, Masahiro Ishikane, Yukumasa Kazuyama, Masato Ikeda, Toyohisa Kondo, Norio Ohmagari, Wataru Sugiura

**Affiliations:** 1 Japan Institute for Health Security, Japan; 2 SB Coronavirus Inspection Center Corp, Japan; 3 Tokyo Metropolitan Government Bureau of General Affairs, Japan; 4 University of Antwerp, Antwerp, Belgium

**Keywords:** case isolation, Japan, pre-boarding PCR test, remote islands, SARS-CoV-2

## Abstract

We assessed the association between voluntary polymerase chain reaction testing of travellers and reported infection rates in the Ogasawara Islands compared with those in several other Tokyo islands. The implementation of polymerase chain reaction testing over a 2-year period was evaluated. Between September 2020 and September 2022, 38,943 of 45,900 travellers to the Ogasawara Islands underwent pre-travel polymerase chain reaction testing, with a notable increase in uptake during states of emergency. Ogasawara reported 385 positive coronavirus disease 2019 cases, with no hospitalizations or severe cases among residents, in contrast to the higher infection and hospitalization rates in Tokyo. Pre-boarding polymerase chain reaction tests were associated with lower reported infection rates in this island setting. These findings suggest that combining pre-travel testing with local mitigation measures, including case isolation systems, may help safeguard the communities of small, geographically isolated islands. These results may inform public health preparedness and response strategies for future infectious disease outbreaks.

## Introduction

Severe acute respiratory syndrome coronavirus 2 (SARS-CoV-2), also known as coronavirus disease (COVID-19), was first identified in Wuhan, China, in late 2019 and rapidly spread worldwide [1]. Governments implemented travel regulations and public health measures, including entry screening, masking, physical distancing, and lockdowns, which helped mitigate regional transmission [2,3]. Remote and isolated regions faced particular challenges during the pandemic, highlighting the need for context-specific, targeted preventive measures. Remote Japanese islands are especially vulnerable to infectious diseases owing to limited healthcare infrastructure and geographical isolation, amplifying the potential impact of outbreaks.

Comparable challenges were observed elsewhere. In Mali, authorities struggled to contact positive cases, lacked isolation facilities, and reported behavioural changes among displaced populations [3]. In contrast, many US-affiliated Pacific Islands implemented early and strict border and quarantine measures, which helped delay community transmission, allowing time to establish testing capacity and achieve high immunization coverage. Guam and the Commonwealth of the Northern Mariana Islands maintained open borders but enforced mandatory quarantine protocols for arriving travellers, which also contributed to delaying widespread transmission during the early stages of the pandemic [4]. Primary strategies to prevent COVID-19 transmission included vaccination, travel restrictions, and diagnostic testing [5]. Polymerase chain reaction (PCR) testing, a cornerstone of SARS-CoV-2 detection [6], enables the rapid identification of infected individuals for isolation, which may help limit viral spread. Despite its recognized utility, the application of PCR testing in managing or monitoring COVID-19 transmission on remote islands remains understudied [7].

Although extensive research has focused on COVID-19 prevention, a significant gap exists in the evaluation of voluntary PCR testing among travellers to remote Japanese islands. Few studies have addressed the unique challenges faced by these islands, leaving key uncertainties regarding vulnerability and potential consequences. In a global context where the effectiveness of public health strategies is paramount, this study is particularly relevant for safeguarding vulnerable populations. Therefore, we examined the implementation and outcomes of voluntary pre-boarding PCR testing among travellers to remote Japanese islands and explored its association with reported infection trends, aiming to provide evidence to inform future infectious disease preparedness.

## Methods

### Study population and sample collection protocol

The Ogasawara Islands are located approximately 1,000 km from mainland Japan and are accessible only by a weekly 24-h ferry service. This study included travellers aged ≥6 years going from the mainland to Ogasawara; returnees were excluded. Participants voluntarily submitted saliva samples to the SB Coronavirus Inspection center Corp. the day before travel.

### Case determination and data collection

Travellers received ZEESAN Saliva RNA Sample Collection Kits. Samples collected 24–96 h before departure were analysed at the SB Coronavirus Inspection center Corp. (Tokyo, Japan) using the SARS-CoV-2 Direct Detection RT-qPCR Kit (Takara Bio Inc., Shiga, Japan) according to the manufacturer’s instructions. Positive results (Ct < 40) led to travel prohibition and referral for medical care. COVID-19 case data for Tokyo and other islands were obtained from official websites [8–14]. Entry restrictions and control measure data were obtained from the authorities and shipping companies. No pre-departure tests were conducted on other islands.

### Descriptive epidemiology

This observational study utilized a survey using data derived from a testing programme implemented as part of Tokyo’s infection control policies. Ethics approval allowed the secondary use of data with online disclosure and opt-out. Demographic and health data (age, sex, current diseases, vaccination history, and testing awareness) were collected via a questionnaire. Disease and attitude data were collected from May 2021 to September 2022; vaccination history was collected from May to September 2022; the average stay was 1.5–4.0 days, based on a major Japanese travel agency itinerary [15–17].

### Ethics approval

The procedures complied with national and institutional committees on human experimentation and with the Declaration of Helsinki (1975, revised in 2008). Sample collection ensured anonymity. Study details and opt-out information were provided on the hospital website. For minors, refusals from the participant or guardian were honoured. The study was approved by the Institutional Review Board of the National center for Global Health and Medicine (approval number: NCGM-G-003678-00; 5 August 2020).

### Statistical analysis

Descriptive statistics are presented as medians (interquartile ranges) or counts (percentages). Incidence rates per 1,000 person-days were calculated for each island and study period. We implemented an island-by-period panel comparison, focusing on three predefined intervals reflecting policy and epidemiological contexts: (1) the entire study period (September 2020–September 2022), (2) the pre-Omicron phase (September 2020–May 2022), and (3) the 7th wave dominated by the Omicron BA.5 lineage (June–September 2022). For each interval, the incidence rate ratio (IRR) for each island was calculated using Ogasawara as a reference, and the corresponding 95% confidence interval (CI) was calculated assuming a Poisson distribution. The testing rate ratio and corresponding 95% CI values were estimated using a modified Poisson regression model [18]. Statistical analyses were performed using SPSS (version 25; IBM Corp., Armonk, NY, USA) or R version 4.3.1 (R Foundation for Statistical Computing, Vienna, Austria).

## Results

### Characteristics of travellers

Between September 2020 and September 2022, 45,900 individuals travelled to the Ogasawara Islands. Of these, 38,943 (86.7%) eligible individuals aged ≥6 years willingly provided specimens and participated in the questionnaire survey (Table 1). The median age of the travellers was 43 years, with 5,187 (13.3%) aged ≥65 years, indicating an elevated risk of severe illness. The sex distribution was 61% male and 38.4% female, with travel motives including tourism (56.2%), business (23.1%), and residence (13.4%). Notably, 13.6% had health conditions that posed potential risks. From May to September 2022, 85.4% of the participants were vaccinated.

**Table 1.** Demographic and general characteristics of travellers to Ogasawara

Variable	Entire period^a^ N (%)	2022/6–2022/9 The 7th wave in Japan N (%)	Positive pre-boarding qPCR test N (%)
Total number of passengers who boarded the ferry^b^	45,900	11,104	—
Total number of individuals considered for pre-boarding test^c^	44,927	10,825	—
Total number of persons who submitted specimens for analysis^d^	38,943/44,927 (86.7)	8,866/10,825 (81.9)	—
Total number of persons who submitted specimens for analysis^d^ ≥ 65 years	5,187/38,943 (13.3)	1,117/8,866 (12.6)	10/148 (6.8)
Age (years), median (IQR)^e^	43 (29–56)	43 (28–56)	38 (26–49)
Sex			
Male	24,202/39,656 (61.0)	5,142/8,939 (57.5)	90/148 (60.8)
Female	15,215/39,656 (38.4)	3,734/8,939 (41.8)	58/148 (39.2)
Unknown	239/39,656 (0.6)	63/8,939 (0.7)	0/148 (0.0)
Body temperature (°C) at specimen collection, median (IQR)	36.3 (36.1–36.5)	36.3 (36.2–36.5)	36.4 (36.2–36.6)
Symptoms^f^	222/39,656 (0.6)	41/8,939 (0.5)	11/148 (7.4)
Cough and phlegm	194/39,656 (0.5)	36/8,939 (0.4)	11/148 (7.4)
Fatigue	40/39,656 (0.1)	12/8,939 (0.1)	4/148 (2.7)
Olfactory impairment	19/39,656 (0.1)	6/8,939 (0.1)	0/148 (0.0)
Dysgeusia	18/39,656 (0.1)	6/8,939 (0.1)	0/148 (0.0)
Others	54/39,656 (0.1)	41/8,939 (0.5)	5/148 (3.4)
No symptoms	38,787/39,656 (97.8)	8,775/8,939 (98.2)	136/148 (91.9)
Unknown/missing	647/39,656 (1.6)	123/8,939 (1.4)	1/148 (0.7)
Reasons for visiting the island^f^, n (%)			
Residence	5,309/39,656 (13.4)	1,098/8,939 (12.3)	32/148 (21.6)
Returning home	1,821/39,656 (4.6)	394/8,939 (4.4)	13/148 (8.8)
Business	9,154/39,656 (23.1)	1,289/8,939 (14.4)	23/148 (15.5)
Tourism	22,284/39,656 (56.2)	5,951/8,939 (66.6)	77/148 (52.0)
Others	1,368/39,656 (3.5)	244/8,939 (2.7)	3/148 (2.0)
Unknown/missing	90/39,656 (0.2)	24/8,939 (0.3)	0/148 (0)
Diseases currently under treatment^f^			
Diabetes	875/29,176 (3.0)	237/8,939 (2.7)	0/141 (0.0)
Heart disease	249/29,176 (0.9)	82/8,939 (0.9)	0/141 (0.0)
Chronic obstructive pulmonary disease	22/29,176 (0.1)	5/8,939 (0.1)	0/141 (0.0)
Chronic kidney disease	71/29,176 (0.2)	25/8,939 (0.3)	0/141 (0.0)
High blood pressure	2,731/29,176 (9.4)	803/8,939 (9.0)	9/141 (6.4)
Others	2,903/29,176 (9.9)	7,214/8,939 (80.7)	11/141 (7.8)
Unknown/missing	14,160/29,176 (48.5)	132/8,939 (1.5)	34/141 (24.1)
History of vaccination			
Yes	8,530/9,988 (85.4)	7,618/8,939 (85.2)	71/95 (74.7)
No	1,396/9,988 (14.0)	1,276/8,939 (14.3)	24/95 (25.3)
No answer	62/9,988(0.6)	45/8,939 (0.5)	0/95 (0.0)
Time to saliva collection	7.5 (3.0–10.0)	7.3 (3.0–10.0)	8.0 (3.0–10.0)
Feel safe to visit the island			
Strongly agree	10,660/29,176 (36.5)	3,011/8,939 (33.7)	41/141 (29.1)
Agree a little	13,161/29,176 (45.1)	4,056/8,939 (45.4)	63/141 (44.7)
Neither agree nor disagree	4,580/29,176 (15.7)	1,655/8,939 (18.5)	36/141 (25.5)
Disagree a little	398/29,176 (1.4)	110/8,939 (1.2)	1/141 (0.7)
Strongly disagree	145/29,176 (0.5)	44/8,939 (0.5)	0/141 (0.0)
No answer	232/29,176 (0.8)	63/8,939 (0.7)	0/141 (0.0)
Ease of testing			
Very easy	5,348/17,353 (30.8)	—	11/37 (29.7)
Easy	7,948/17,353 (45.8)	—	17/37 (45.9)
Neither	2,364/17,353 (13.6)	—	8/37 (21.6)
Difficult	1,040/17,353 (6.0)	—	1/37 (2.7)
Very difficult	194/17,353 (1.1)	—	0/37 (0.0)
No answer	459/17,353 (2.6)	—	0/37 (0.0)
Necessity for testing			
Strongly agree	14,698/29,176 (50.4)	3,213/8,939 (35.9)	62/141 (44.0)
Agree a little	10,203/29,176 (35.0)	3,850/8,939 (43.1)	60/141 (42.6)
Neither agree nor disagree	2,955/29,176 (10.1)	1,265/8,939 (14.2)	13/141 (9.2)
Disagree a little	609/29,176 (2.1)	335/8,939 (3.7)	2/141 (1.4)
Strongly disagree	485/29,176 (1.7)	214/8,939 (2.4)	2/141 (1.4)
No answer	226/29,176 (0.8)	62/8,939 (0.7)	2/141 (1.4)

a The information on diseases under treatment and attitudes towards testing was collected between May 2021 and September 2022, and that on vaccination history was collected only between May 2022 and September 2022.

b Refers to the actual number of passengers onboard, including those aged <6 years.

c All prospective passengers, except those aged <6 years, were considered for testing.

d Indicates the number of specimens actually received from individuals subject to testing. Passengers who cancelled boarding or were not allowed to board because of positive screening test results were included. Passengers who cancelled for other reasons were excluded.

e IQR, interquartile range.

f When counting ‘Symptoms’, ‘Reason for Visit’, and ‘Currently Treated Disease’, multiple answers were accepted.

qPCR, quantitative polymerase chain reaction.

### Infection trends in Ogasawara

**Figure 1.**
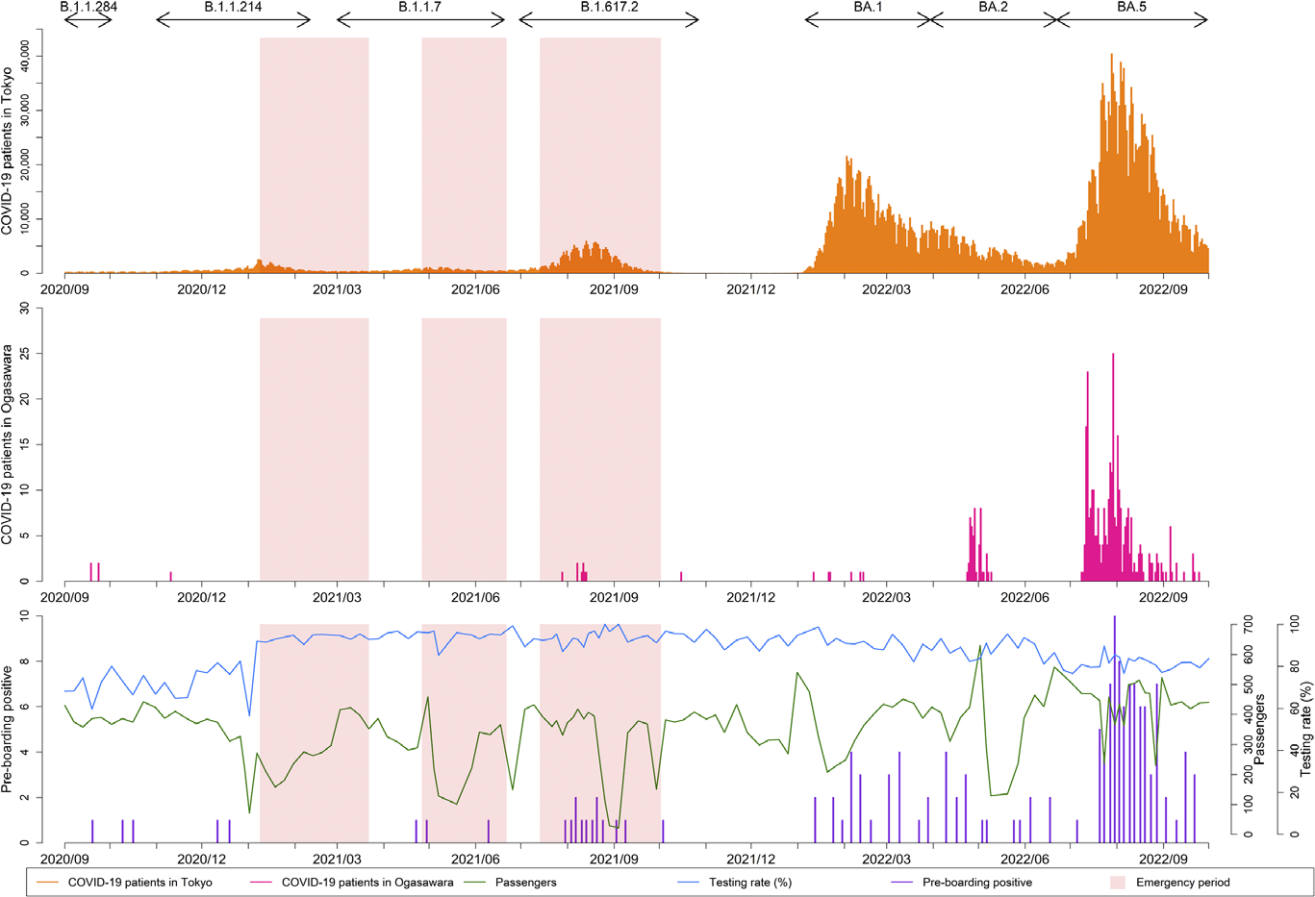
Patients with COVID-19 in Ogasawara and Tokyo. The pink bars indicate COVID-19 patients in Ogasawara, and the purple bars indicate the individuals who tested positive for COVID-19 before boarding to Ogasawara between 1 September 2020 and 30 September 2022. The green line indicates the number of passengers travelling from Tokyo to Ogasawara, and the blue line indicates the testing rate of saliva samples using PCR before boarding the vessel. Orange bars indicate COVID-19 cases in Tokyo between 1 September 2020 and 30 September 2022. The period of the declaration of the state of emergency is indicated by pink blocks. The main prevalent variants are indicated by arrows. COVID-19: coronavirus disease.

During the study period (1 September 2020–30 September 2022), Ogasawara Village reported 385 positive cases, of which 44 involved individuals aged >60 years (Figure 1). The highest daily caseload in Ogasawara, 25 cases, was recorded on 29 July 2022. In contrast, Tokyo reported a total of 3,147,331 cases across Waves 2–7, leading to 1,481,307 (47.1%) hospitalizations and 41,947 (1.3%) severe cases. Ogasawara Village reported neither hospitalizations nor severe cases during the same period.

**Figure 2.**
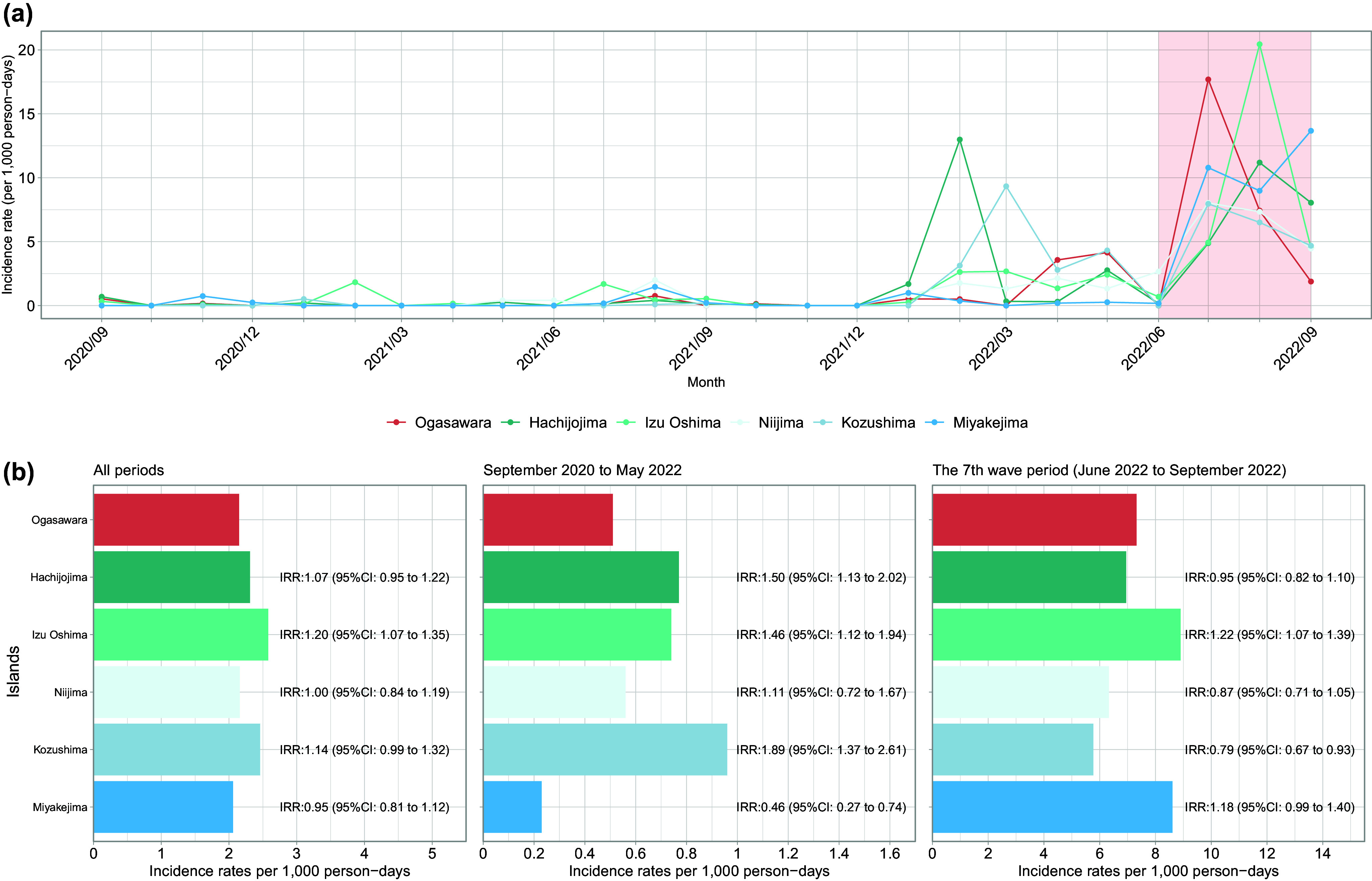
(a) Temporal trends in incidence rates of COVID-19 on the Tokyo islands, calculated based on the number of days visitors stayed on each island. (b) Comparison of incidence rates per 1,000 person-days for Ogasawara and other Tokyo islands during three periods: the entire study period (September 2020–September 2022), the pre-Omicron phase (September 2020–May 2022), and the 7th wave dominated by the Omicron BA.5 lineage (June–September 2022). Each bar indicates the infection rate per 1,000 person-days. The left, middle, and right figures show the infection rates for all periods from September 2020 to May 2022 and from June 2022 to September 2022, respectively. The IRR for each island was calculated using Ogasawara as a reference, and the corresponding 95% confidence interval (95% CI) was calculated by assuming a Poisson distribution. IRR: incidence rate ratio; CI: confidence interval.

Monthly incidence rates per 1,000 person-days were calculated for Ogasawara and other Tokyo islands to visualize temporal fluctuations (Figure 2a). Distinct seasonal and wave-specific patterns were observed, with Ogasawara showing consistently low rates, except during the nationwide 7th wave (June–September 2022).

IRRs (cases per 1,000 person-days) were estimated for three predefined periods: the entire study period (September 2020–September 2022), the pre-Omicron phase (September 2020–May 2022), and the 7th wave dominated by Omicron BA.5 (June–September 2022) (Figure 2b, Table S1). During the pre-Omicron phase, all islands except Niijima and Miyakejima showed significantly higher IRRs than Ogasawara, whereas the differences diminished during the 7th wave.

### Pre-boarding qPCR testing

**Figure 3.**
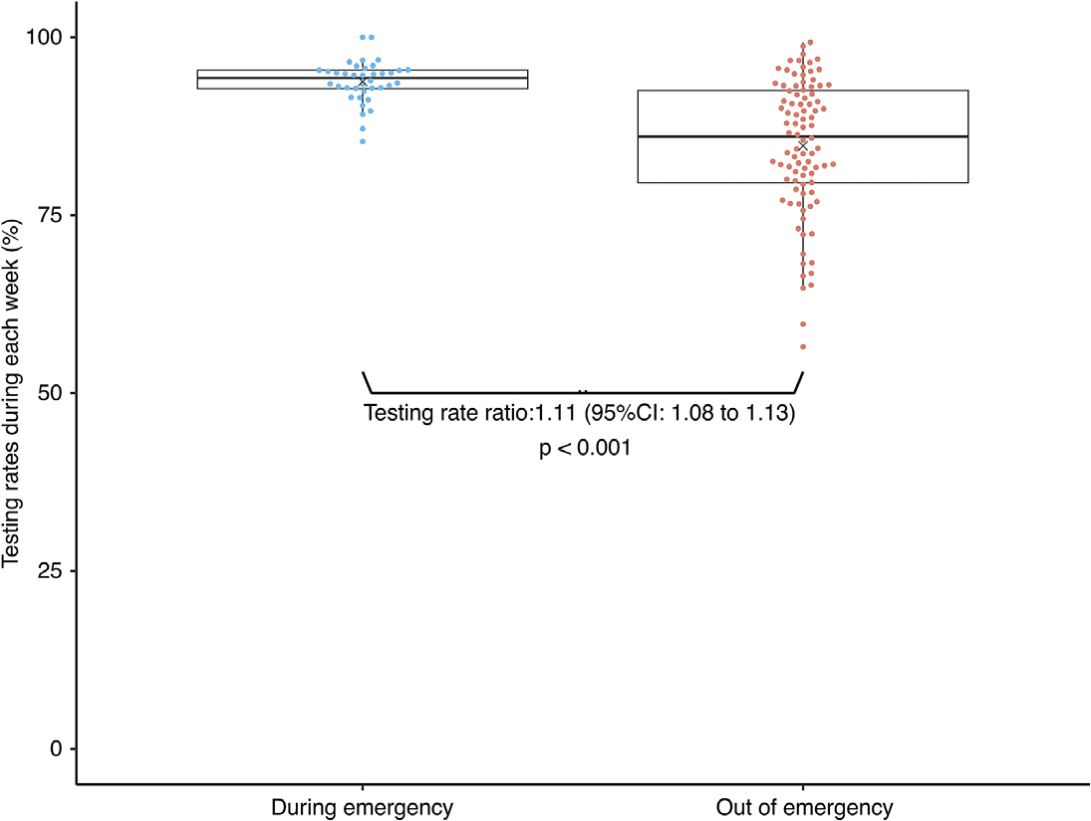
Boxplot of testing rates during and after the Japanese government declared a state of emergency in response to the surge in coronavirus infections. The orange and blue circles indicate the testing rate for each week using PCR before boarding the vessel, and the × mark indicates the mean testing rate.

From 1 September 2020 to 30 September 2022, 38,943 individuals underwent pre-travel PCR testing, representing 86.7% of those eligible (Table 1). Test participation increased to 93.8% during the emergency declaration period, compared with 84.7% in the remainder of the study (p < 0.001; Figure 3). Among all individuals who underwent testing during the study period, 148 who tested positive and did not travel (Figure 1) were not followed up. The median body temperature at the time of saliva collection was 36.3 °C, and 97.8% of those tested were asymptomatic (Table 1). Symptomatic cases included cough and phlegm (0.5%), fatigue (0.1%), olfactory impairment (0.1%), and dysgeusia (0.1%). Although most individuals were asymptomatic, those with positive pre-boarding test results had a higher proportion of symptoms (10.8%), particularly coughing and phlegm expression (7.4%). Most respondents reported that the test was easy to perform, necessary, and reassuring for safe travel.

### Comparative analysis

In this island-by-period panel comparison, Ogasawara, where pre-departure testing was implemented, generally maintained lower incidence rates than several other comparison islands (Figure 2b, Table S1). These findings suggest an association between widespread testing uptake and the lower incidence rates observed in Ogasawara. However, the observational design did not allow causal inference regarding subsequent community transmission.

## Discussion

We investigated the association between voluntary pre-boarding PCR testing among travellers to Ogasawara and reported island-level infection trends from September 2020 to September 2022. We observed that periods with higher uptake of pre-boarding PCR testing were associated with lower reported infection rates in Ogasawara compared with those in several other Tokyo islands. Because this was an observational study conducted in the presence of multiple concurrent public health measures, causal relationships cannot be inferred.

The high testing rate of 86.7% over the entire period, particularly in emergency situations (93.8%), emphasizes the feasibility and acceptability of this preventative measure. Testing is an important component of COVID-19 control, yet barriers and hesitancy can affect participation rates [19]. Local government coverage of test costs, combined with the use of self-collected saliva samples, likely enhanced acceptance. The same-day delivery of results by private companies may also have increased uptake.

Fortunately, 80% of the individuals who contracted COVID-19 in 7th Wave had already received two doses of the vaccine. Most cases during the Omicron-dominant period were mild, and no severe disease was reported in Ogasawara.

In Ogasawara, pre-boarding PCR testing was voluntary; thus, some of the 14% of travellers who did not undergo PCR testing prior to travel may have been infected. Schneitler et al. reported similarly low SARS-CoV-2 positivity in Germany during high transmission, partly due to travellers’ self-isolation 7–10 days before testing to avoid trip cancellations and costs [20]. Such behaviour may have occurred in Ogasawara; however, voluntary testing likely missed some infections, especially during surges in Tokyo. Schneitler et al. further noted that mandatory testing aids in detection and engages traveller groups otherwise unlikely to seek pre-travel medical advice – benefits not realized in Ogasawara’s voluntary system [20].

In 7th Wave, 77.08% of the Japanese population had completed a second vaccine dose as of 2 July 2022; however, the third-dose rate was still low at 62.60% and may have been even lower among younger people [21]. The average age of travellers was 46 years. Individuals who had received the second vaccination and believed themselves not to have COVID-19 might still have been infected. However, voluntary testing may result in missing infections, especially among asymptomatic individuals or during surges, and the potential advantages of mandatory testing – such as broader and more uniform coverage across traveller groups – were not realized in this setting.

Notably, no hospitalizations or severe cases were reported in Ogasawara despite the substantial number of travellers. During the pandemic, medical facilities initially transported patients with COVID-19 to Tokyo by helicopter. Subsequently, individuals with mild disease were managed through home- or accommodation-based isolation to avoid unnecessary hospitalization. Rapid PCR testing after symptom onset and voluntary isolation systems may have aided timely case management. These experiences illustrate how the Ogasawara healthcare system adapted to the constraints of a remote island setting during the COVID-19 pandemic. Local antigen and PCR testing were important for rapid identification and isolation, representing an adaptive approach to patient management. Unlike the previous practice of transporting patients to Tokyo, the revised strategy emphasized home or facility isolation of mild cases, optimizing local resources.

Even during the pandemic, tourism (56.2%) was the main reason for travel, followed by business (23.1%), residence (13.4%), and returning home (4.6%). The local government implemented several measures to mitigate transmission risk. Residents and tourists were urged to refrain from unnecessary outings after 8:00 PM. To enhance safety, individuals who underwent pre-travel testing were provided with wristbands as visible proof of testing. Tourist facilities prioritized those wearing wristbands, and restaurants adjusted operational hours to reduce service times. Vessel capacity, the exclusive mode of transport to Ogasawara, was restricted by 30–50% to ensure passenger spacing. These measures were implemented concurrently to reduce transmission risk while sustaining essential economic activity, and their individual effects could not be elucidated in this analysis.

The geographical context of Ogasawara – located approximately 1,000 km from mainland Japan and accessible only by a weekly 24-h ferry – provides a unique setting for examining infection trends (Supplementary Figure S1). Travellers spend 2–3 days longer on Ogasawara than on other Tokyo metropolitan islands. Despite longer stays, the infection rates in Ogasawara were similar to or lower than those in other islands. In particular, during the pre-Omicron phase (September 2020–May 2022), IRRs for all islands, except Miyakejima and Niijima, were significantly higher than those for Ogasawara, whereas differences diminished during the 7th wave (June–September 2022).

Several limitations of the study should be considered. It was observational, and we could not control for concurrent public health measures, behavioural changes, or vaccination trends. In addition, outbreak seeding and sustained transmission in small island populations can be highly stochastic, and the observed incidence is strongly influenced by chance contact patterns and low absolute case numbers. Saliva samples were self-collected, potentially increasing the risk of improper collection and false-negative PCR results. The sensitivity and specificity of the saliva test were 86.4% (95% CI: 82.8–89.4%) and 97.0% (95% CI: 95.0–98.3%), respectively, which could result in false negatives during early infection [22]. Previous studies indicate that increasing screening frequency is more effective than improving test accuracy in preventing infection [23]. More detailed information on the infection status of travellers after arrival is required to rigorously evaluate the association between pre-boarding testing and island-level transmission. Additionally, the healthcare system may not have identified all COVID-19 cases. Travellers were advised to undergo PCR testing prior to travel and to wear wristbands as proof, suggesting that they acted cautiously while on the island. Although details of household composition were unavailable, close household contact may have facilitated local transmission. Such contextual data would enable more refined analysis and inform infection control measures. Future studies should consider the quantitative evaluation of vaccination efficacy and cost-effectiveness, as well as the behavioural context of testing.

In conclusion, voluntary pre-boarding PCR screening was associated with lower reported infection rates in Ogasawara during the study period. However, causal relationships could not be established owing to the observational study design, concurrent interventions, and the stochastic nature of transmission in small populations. Nevertheless, the findings underscore the potential value of integrated approaches that combine accessible testing, vaccination, and adaptive local healthcare systems in preparing for future infectious disease threats in remote settings.

## Supporting information

10.1017/S0950268826101289.sm001 Kimura et al. supplementary material Kimura et al. supplementary material

## Data Availability

Not applicable.

## Supplementary material

The supplementary material for this article can be found at http://doi.org/10.1017/S0950268826101289.

## References

[1] Mallapaty S (2020) What the cruise-ship outbreaks reveal about COVID-19. Nature 580, 18. 10.1038/d41586-020-00885-w. 32218546

[2] Chinazzi M, et al. (2020) The effect of travel restrictions on the spread of the 2019 novel coronavirus (COVID-19) outbreak. Science 368, 395–400. 10.1126/science.aba9757. 32144116 PMC7164386

[3] Gwee SXW, et al. (2021) Impact of travel ban implementation on COVID-19 spread in Singapore, Taiwan, Hong Kong and South Korea during the early phase of the pandemic: A comparative study. BMC Infectious Diseases 21, 799. 10.1186/s12879-021-06449-1. 34380452 PMC8355580

[4] Cash McGinley HL, et al. (2023) COVID-19 in the US-affiliated Pacific islands: A timeline of events and lessons learned from March 2020–November 2022. PLOS Global Public Health 3, e0002052. 10.1371/journal.pgph.0002052. 37585385 PMC10431600

[5] Girum T, et al. (2021) Optimal strategies for COVID-19 prevention from global evidence achieved through social distancing, stay at home, travel restriction and lockdown: A systematic review. Archives of Public Health 79, 150. 10.1186/s13690-021-00663-8. 34419145 PMC8380106

[6] Hay JA, et al. (2021) Estimating epidemiologic dynamics from cross-sectional viral load distributions. Science 373, eabh0635. 10.1126/science.abh0635. 34083451 PMC8527857

[7] Terada-Hirashima J, et al. (2022) Investigation of the use of PCR testing prior to ship boarding to prevent the spread of SARS-CoV-2 from urban areas to less-populated remote islands. Global Health & Medicine 4, 174–179. 10.35772/ghm.2022.01008. 35855067 PMC9243411

[8] Ogasawara Village https://en.vill.ogasawara.tokyo.jp (accessed 6 February 2023).

[9] Hachijo Town https://www.town.hachijo.tokyo.jp (accessed 2 December 2022).

[10] Oshima Town (https://www.town.oshima.tokyo.jp (accessed 2 December 2022).

[11] Niijima Village https://www.niijima.com/index.html (accessed 2 December 2022).

[12] Kouzushima Village https://www.vill.kouzushima.tokyo.jp (accessed 2 December 2022).

[13] Miyake Village https://www.vill.miyake.tokyo.jp (accessed 2 December 2022).

[14] Office of the Governor for Policy Planning. Efforts across the Tokyo Metropolitan Government. https://www.seisakukikaku.metro.tokyo.lg.jp/cross-efforts/corona/torikumi (accessed 20 December 2022).

[15] Club Tourism https://www.club-t.com (accessed 20 October 2023).

[16] *JTB* https://www.jtb.co.jp (accessed 20 October 2023).

[17] HIS https://www.his-j.com/Default.aspx (accessed 20 October 2023).

[18] Zou G (2004) A modified Poisson regression approach to prospective studies with binary data. American Journal of Epidemiology 159, 702–706. 10.1093/aje/kwh090. 15033648

[19] Embrett M, et al. (2022) Barriers to and strategies to address COVID-19 testing hesitancy: A rapid scoping review. BMC Public Health 22, 750. 10.1186/s12889-022-13127-7. 35422031 PMC9008387

[20] Schneitler S, et al. (2021) Experiences with pre-travel diagnostic PCR testing for SARS-CoV-2: Challenges and opportunities. Journal of Travel Medicine 28, taab116. 10.1093/jtm/taab116. 34323275

[21] Digital Agency, Government of Japan. In the original version of this paper, the reference was made to a webpage that is no longer accessible. As of 2 July, 2022, the content was available at https://info.vrs.digital.go.jp/dashboard://info.vrs.digital.go.jp/dashboard/resource. The webpage has since been removed or is no longer accessible. (https://info.vrs.digital.go.jp/dashboard). (accessed 2 July 2022).

[22] Zhu J, et al. (2020) Viral dynamics of SARS-CoV-2 in saliva from infected patients. Journal of Infection 81, e48–e50. 10.1016/j.jinf.2020.06.059. 32593658 PMC7316041

[23] Kucirka LM, et al. (2020) Variation in false-negative rate of reverse transcriptase polymerase chain reaction-based SARS-CoV-2 tests by time since exposure. Annals of Internal Medicine 173, 262–267. 10.7326/M20-1495. 32422057 PMC7240870

