# Supplementary material for: Effectiveness of voluntary PCR testing against COVID-19 spread in remote Japanese islands
Source: Epidemiol Infect. 2026 Mar 24;154:e48. doi: 10.1017/S0950268826101289 (PMC13100917; doi:10.1017/S0950268826101289)
Supplement: Kimura et al. supplementary material [file S0950268826101289sup001.docx]

**Supplementary Materials**

**Table S1**. Incidence rates on the islands of Tokyo

|  | All periods (Sep 2020–Sep 2022) | | | Pre-Omicron period (Sep 2020–May 2022) | | | The 7th infectious wave (June 2022–Sep 2022) | | |  |
| --- | --- | --- | --- | --- | --- | --- | --- | --- | --- | --- |
|  | Rate per 1000 person-days | Rate ratio (95% CI) | p-value | Rate per 1000 person-days | Rate ratio (95% CI) | p-value | Rate per 1000 person-days | Rate ratio (95% CI) | p-value | |
| Ogasawara | 2.15 | Ref. | - | 0.51 | Ref. | - | 7.32 | Ref. | - | |
| Hachijojima | 2.31 | 1.07 (0.95–1.22) | 0.282 | 0.77 | 1.50 (1.13–2.02) | 0.004 | 6.95 | 0.95 (0.82–1.10) | 0.499 | |
| Izu Oshima | 2.58 | 1.20 (1.07–1.35) | 0.002 | 0.74 | 1.46 (1.12–1.94) | 0.005 | 8.90 | 1.22 (1.07–1.39) | 0.003 | |
| Niijima | 2.16 | 1.00 (0.84–1.19) | 1.000 | 0.56 | 1.11 (0.72–1.67) | 0.690 | 6.33 | 0.87 (0.71–1.05) | 0.147 | |
| Kozushima | 2.46 | 1.14 (0.99–1.32) | 0.077 | 0.96 | 1.89 (1.37–2.61) | <0.001 | 5.77 | 0.79 (0.67–0.93) | 0.005 | |
| Miyakejima | 2.06 | 0.95 (0.81–1.12) | 0.584 | 0.23 | 0.46 (0.27–0.74) | 0.001 | 8.61 | 1.18 (0.99–1.40) | 0.064 | |

CI: confidence interval.


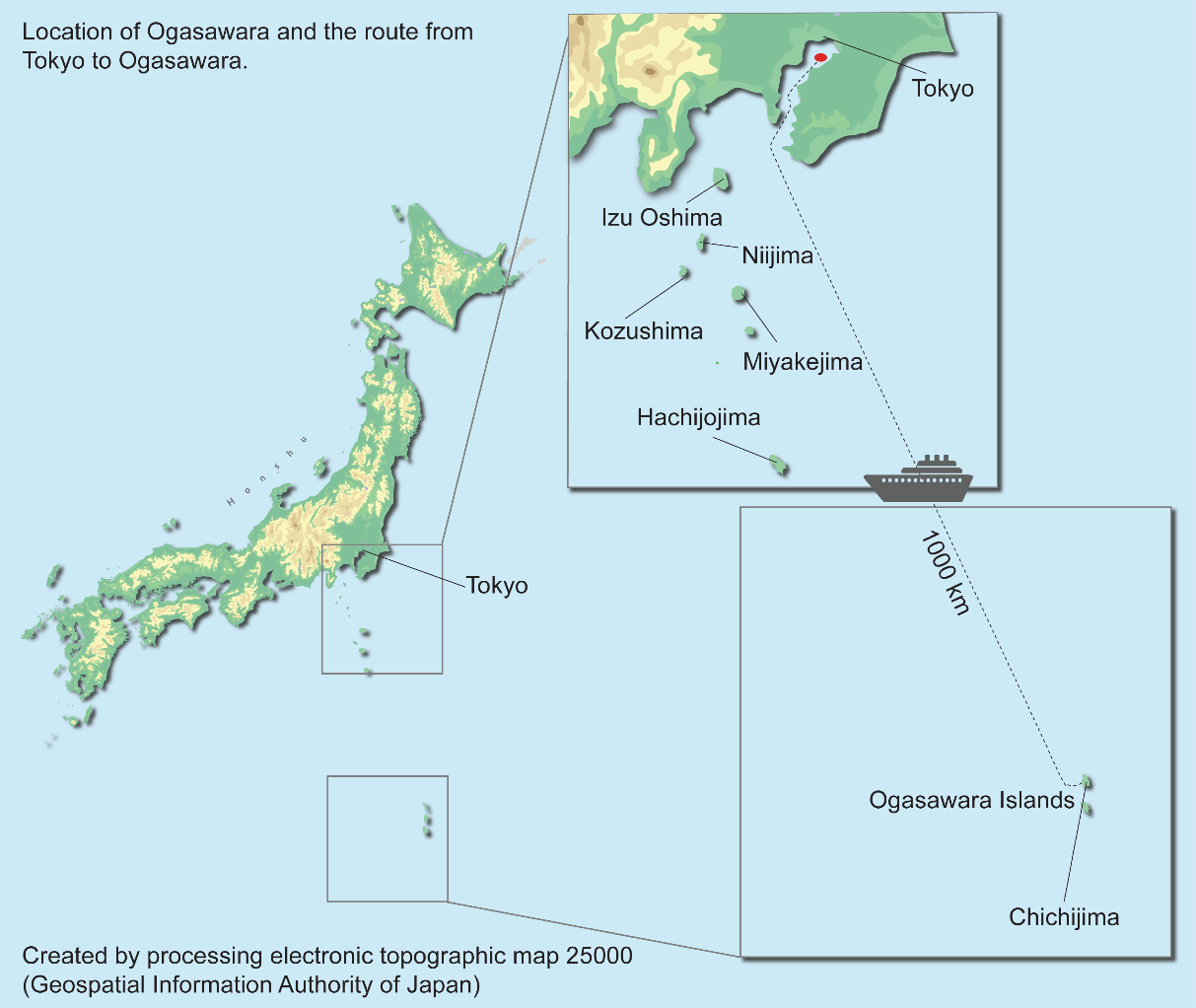


**Figure S1**. Location of Ogasawara and the route from Tokyo to Ogasawara
